# Considerations for Performing Level-2 Centiloid Transformations for Amyloid PET SUVR values

**DOI:** 10.1038/s41598-018-25459-9

**Published:** 2018-05-09

**Authors:** Christopher G. Schwarz, Nirubol Tosakulwong, Matthew L. Senjem, Jeffrey L. Gunter, Terry M. Therneau, Prashanthi Vemuri, Val J. Lowe, Clifford R. Jack

**Affiliations:** 1 Department of Radiology, Mayo Clinic and Foundation, Rochester, MN USA; 2 Department of Health Sciences Research, Division of Biostatistics, Mayo Clinic and Foundation, Rochester, MN USA; 3 Department of Information Technology, Mayo Clinic and Foundation, Rochester, MN USA

## Abstract

The Centiloid Project describes a post-hoc data transformation to standardize amyloid PET measurements to enable direct data comparisons across sites and studies using differing acquisition/analysis methods. It uses linear regression that transforms values using different measurement scales to match those from a standard Centiloid unit scale. Our group’s measurement method differs from the Centiloid’s standard method in both acquisition and analysis methods. In this work we examine multiple variations for performing these transformations and compare several approaches. We hypothesized that using Deming regression, which accounts for error on both axes, would produce a more optimal transformation than the recommended standard linear regression. We also examined the effects of performing separate regressions for differences in acquisition and analysis methods, rather than a direct single-regression approach. Our results found that all transformation approaches had very similar performance and were within the recommended tolerance thresholds.

## Introduction

Many groups employ amyloid Positron Emission Tomography (PET) imaging in their research programs. However, different image acquisition sites and analysis groups use different tracers (with different binding properties) and different quantification software methods, all of which influence the scales of quantitative measurements. As a result, data values cannot be directly compared across sites. This issue also confounds meta-analyses across studies and hinders the creation of field-standardized biomarkers and thresholds.

The Centiloid scale was proposed as a method to harmonize amyloid PET measurements across differing acquisition methods/tracers and image processing methods by performing a series of linear regressions to convert them to a standardized 0–100 scale^1^. The Centiloid standard scale was defined with images using the [11 C] Pittsburgh Compound B (PiB) tracer^2^, measured by late-uptake Standardized Uptake Value Ratio (SUVR) images acquired between 50–70 minutes post-injection (PiB_50–70_), and a software pipeline based on the Statistical Parametric Mapping version 8 (SPM8) package^3^ with a specific set of brain atlas volumes of interest (VOIs).

According to the Centiloid approach, imaging/analysis sites using this specific combination of acquisition and analysis parameters can convert values to Centiloid units by using a standard, published “level-1” equation. Sites where these parameters differ (i.e. different acquisition and/or processing steps) must perform “level-2” Centiloid transformations to determine the linear regression equations to transform data by correcting for differences in acquisition/processing methods between their measurements and Centiloid-standard measurements. Follow-up abstracts and manuscripts have described the results of this process for the NAV4694^4^, Florbetaben^5^ and Flutemetamol^6^ tracers.

In this work we detail our analyses to determine an optimal transformation from our Mayo PiB SUVR method to the Centiloid Standard PiB SUVR method. The Mayo method differs from the standard method in its acquisition (PiB acquired from 40–60 minutes post-injection, rather than 50–70 minutes post-injection) and in its image processing approaches^1^. The Centiloid manuscript prescribes a specific approach for performing such transformations. Our objective was to examine this approach and compare it with several modifications in order to identify an optimal approach. When transforming SUVR values from a non-standard method to the reference standard method, the error in this transformation adds to the width of a confidence interval around each transformed measurement in the standard scale. Therefore, a potential reduction of the error in these transformations would directly improve the precision of reported CL data and power of multi-site studies and meta-analyses made possible using Centiloids.

To determine an optimal approach for level-2 Centiloid transforms, we compare three different approaches (described later) for performing the necessary regression analyses. We then compare the resulting transformation equations from each approach and the magnitude of the differences between SUVR values transformed using each approach’s equations.

## Methods

### Subject and Scan Characteristics

The standard Centiloid scale is defined relative to a standard, published set of scans acquired between 50–70 minutes after injection of the PiB tracer. These scans are publicly available at the Centiloid project website http://www.gaain.org/centiloid-project. Characteristics of the subjects and acquisitions used in this dataset have been previously published^1^.

### Image Preprocessing

To use the Centiloid scale, a site using a tracer other than PiB must acquire a cross-over set of scans of subjects imaged with both PiB and their chosen tracer, using previously-described techniques^1^. Cross-over datasets for a subset of available tracers (currently NAV4694, Florbetaben, and Flutemetamol) are also available on the Centiloid project website, which can be used when applicable in lieu of acquiring new cross-over datasets for these tracers. Sites using the Centiloid Standard analysis method with one of these tracers can simply use their accompanying published equations, but those using different analysis methods must follow the prescribed regression steps. Our particular site (Mayo Clinic, Rochester, MN, USA) uses PiB, which matches that of the standard Centiloid dataset, but uses scans acquired 40–60 minutes post-injection, rather than 50–70. Because the Centiloid project website provides dynamic PiB scans acquired over ranges of time including both 40–60 and 50–70 minutes post-injection, we were able to use these standard scans to construct a cross-over dataset to convert between PiB_40–60_ and PiB_50–70_ rather than acquiring a new set of cross-over scans.

To construct a dataset for conversion between PiB_40–60_ and PiB_50–70_, we downloaded the dynamic PiB scan data from the Centiloid project website and converted all scans from DICOM to Nifti using in-house software. The range of acquisition time for each dynamic PET scan frame, relative to the injection time, was determined using the “*(0054,1300) FrameReferenceTime* DICOM header field. For each subject scan, frames occurring between each of the two desired ranges (40–60 minutes and 50–70 minutes) were identified, and the frames within each of the two ranges were voxel-wise averaged to create summed (static) PiB_40–60_ and PiB_50–70_ images. We then used these scans for the analyses described below.

### Processing Methods

In this section, we describe both the Centiloid Standard PiB SUVR Method and our non-standard, Mayo PiB SUVR method. Following sections will then describe the approach used to transform from our non-standard method to the standard method, enabling the use of Centiloid units.

#### Centiloid Standard PiB SUVR Method

The Centiloid “Standard PiB Method” was described in the original Centiloid manuscript^1^, with the purpose of allowing exact replication by other sites. Briefly, SPM8 is used to register each subject’s PiB PET scan to their MRI, and subsequently to register their MRI and PiB PET scans to the MNI152 T1-weighted 2 mm template included with SPM8, using the “Coregister: Estimate” module with default parameters. The “Segment” module in SPM8^7^ is then used on each subject T1-weighted MRI, which combines (nonlinear) spatial normalization to the MNI152 template, bias correction, and tissue segmentation in one step. Normalization parameters produced by this step are applied to each coregistered subject PET and MR images, resampling them to MNI152 template space, using the “Normalise: Write” SPM8 module. Mean values are then calculated in each of the Centiloid standard VOIs (which are defined in MNI152 space) and used to compute SUVR values as the Cortex (CTX) VOI normalized by each of four choices of reference VOIs^2^. When replicating the standard method in this work, we used the VOI definition files provided by the Centiloid project website for both the cortex and the reference VOIs. The process originally used to create them has been previously described^1^.

In this work we use this standard method both via the reference values from the Centiloid project website, and by re-implementing the described steps and running them ourselves. As part of the “replication of level-1 analysis” step in a level-2 analysis, we compared the values from our re-implementation with those of the reference values and verified that the results were within the specified tolerances (see Supplementary Material). For brevity’s sake, we present results in this work using only the whole cerebellum reference VOI, which is considered the standard, rather than all four.

#### Mayo PiB SUVR Method

The Mayo PiB SUVR method used in this work has also been previously described^8^. Briefly, Unified Segmentation in SPM5 is used for bias correction, tissue segmentation, and computation of nonlinear normalization parameters between each image and a standard template. Rather than the standard SPM5 template and tissue probability maps, we use an in-house, population-specific template called “STAND400” that has been previously described^9^. We use SPM5 “Coregister: Estimate” module to register each PET image to its corresponding MRI, and use the “Normalise: Write” module to transform VOIs to the space of the subject MRI, and these VOIs are masked to include only voxels classified as gray matter or white matter by the segmentation. Median values within these masked VOIs are then used to compute SUVR values. The VOIs used in this pipeline were defined in-house on the STAND400 template. The cortical (GlobalPiB) VOI was formed as a combination of voxels in the prefrontal, orbitofrontal, parietal, temporal, anterior cingulate, and posterior cingulate/precuneus regions. Voxels in the cerebellar crus are used as a reference region.

### Glossary of Datasets

In this work, we will discuss five different sets of SUVR values, each computed from the same set of dynamic PET sequences downloaded from the Centiloid project website, using differences in acquisition (PiB_40–60_ vs PiB_50–70_) and processing (Mayo processing pipeline vs. the Centiloid “Standard PiB method” method). These are defined in Table 1.

**Table 1:** Glossary of datasets used in this work.

Name	Processing Method	Acquisition Method
S	Reference SUVR values for each reference PiB scan, from GAAIN.org, using the Centiloid “Standard PiB method”^1^	PiB_50–70_
Ŝ	Values from other methods (below), after application of level-2 transformations to match S	multiple
MS50	Mayo implementation of the Centiloid “Standard PiB Method” using SPM8 and Centiloid standard VOIs^1^ (see Supplementary Material)	PiB_50–70_
MM50	Mayo-internal SPM5 SUVR method using Mayo VOIs^8^	PiB_50–70_
MM40	Mayo-internal SPM5 SUVR method using Mayo VOIs^8^	PiB_40–60_

### Variations in Approaches for Performing Level-2 Centiloid Conversions

In this work we will compare three different approaches for performing level-2 Centiloid conversions. In this section, we describe the two major properties by which these approaches differ from each other.

#### One-Step vs Two-Step Pathways

**Figure 1:**
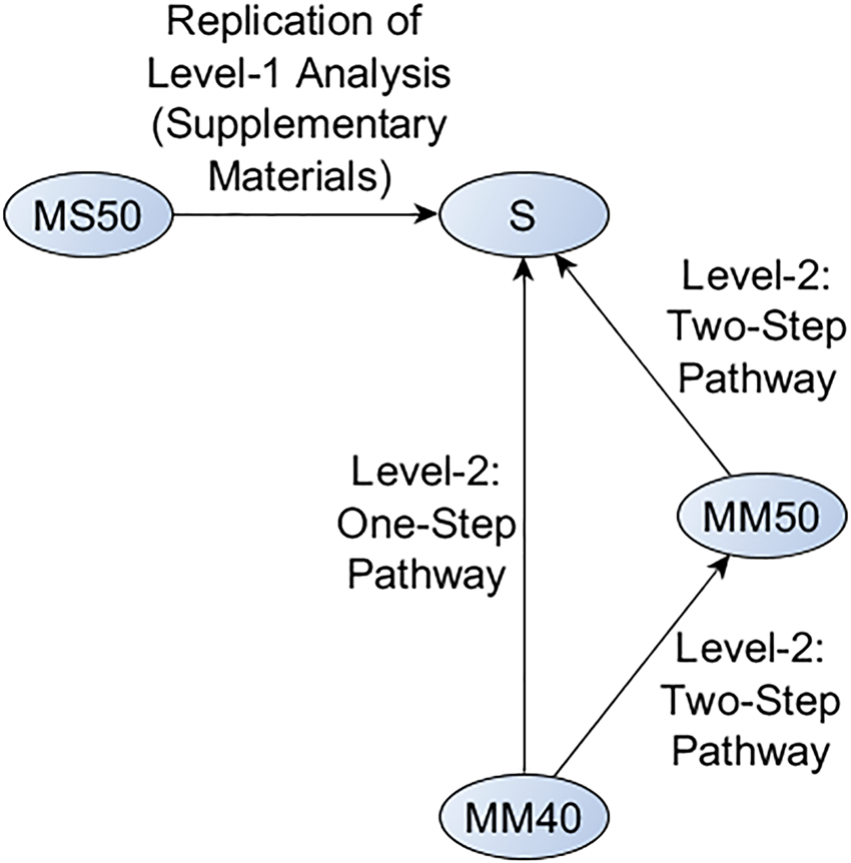
Illustration of the One-Step and Two-Step regression pathways compared in this work.

The Mayo PiB SUVR method differs from the Centiloid Standard method in both acquisition and processing, thus requiring level-2 transformation. The manuscript suggests (equation 2.2.3.2a) that the recommended approach is to perform a single regression between our method with both changes and the standard Centiloid method. For comparison we also examined the effects of performing two separate regressions: first, one for the change in acquisition, and next, one for the change in processing. The final transformation equation for this two-step approach is then a composite of the two transforms. Such a two-step approach may be attractive for sites using F18 tracers with already-published Centiloid conversions, enabling those using different analysis methods to determine a regression to transform between these methods in conjunction with the published equations to transform between tracers. Based on statistical standards for scale calibration, we hypothesized that performing these conversions instead as single transformation may result in less transformation error. An alternate way to think of this is by counting degrees of freedom. Fitting two linear transformations estimates more parameters only to combine them in the final composite transformation. In this work, we quantitatively compare the “Two-Step” regression pathway to the direct “One-Step” pathway. We present a flowchart of these pathways in Fig. 1.

#### Standard Linear Regression vs Deming Regression

The examples in the Centiloid manuscript suggest that the standard method should be used as the predictor variable during level-2 transformations. We generalize this to assume that whichever variable is methodologically nearer to the standard method should be used as the predictor variable. For example, in Fig. 1, MM50 is nearer to S than MM40 is to S because it differs from S in one major aspect rather than two, so MM50 would be used as the predictor variable during the first step in the Two-Step pathway.

An inherent assumption of ordinary linear regression (LR) is that the response variable (y) is subject to error but that the predictor variable (x) is error free (an assumption that is questionable in the present case). Therefore, reversing the choice of predictor vs. response variable would produce slightly different linear regressions. In clinical laboratory work where these types of calibration are an ongoing task (with respect to each new batch of reagent), the standard is to use a regression method that assumes errors in both x and y, e.g. Deming regression (DR). Therefore, we hypothesized that using Deming regression (which produces an identical regression regardless of choice of predictor vs. response variable) would reduce the error in the resulting transformations. Implementations of Deming regression are less widely available than those for standard linear regression, but they have become common enough that we believe its use would not present a substantial technical barrier for research sites performing Centiloid transformations. In this work, we compared one-step and two-step approaches and LR and DR methods to analyze whether these make a substantial difference in the fit, and to determine whether any is more optimal.

### Evaluation Criteria and Statistical Methods

We performed this level-2 Centiloid transformation between MM40 and S using three different variants: one used the two-step approach with standard linear regression, and two used the one-step pathway (one using standard linear regression and one using Deming regression). We list these variants in Table 2. To compare them, we used the percent differences between the MM40 methods, transformed in each of these ways to match S (i.e. in standard Centiloid PiB SUVR units), and the original reference (S) values. We also transformed values from standard Centiloid PiB SUVR to Centiloid (CL) units using Eq. 1.3b from the Centiloid manuscript^1^, to present our comparisons in both unit scales. We present these results in the following section.

**Table 2:** Description of the regression variations compared in this work.

New Name	Type	Step 1	Step 2
Ŝ_1-Step_linear_	standard linear regression	lm(MM40 ~ S)	N/A
Ŝ_1-Step_Deming_	Deming regression	deming(MM40 ~ S)	N/A
Ŝ_2-Step_linear_	standard linear regression; the standard Centiloid project approach	lm(MM40 ~ MM50)	lm(MM50 ~ S)

Regression steps are specified in the R programming language, which uses a method (response ~ predictor) format.

### Data Availability

The numerical datasets generated during and/or analyzed during the current study are available from the corresponding author on reasonable request. The Centiloid PiB dataset that we used to generate these numerical datasets are available publically from http://www.gaain.org/centiloid-project.

## Results

**Figure 2:**
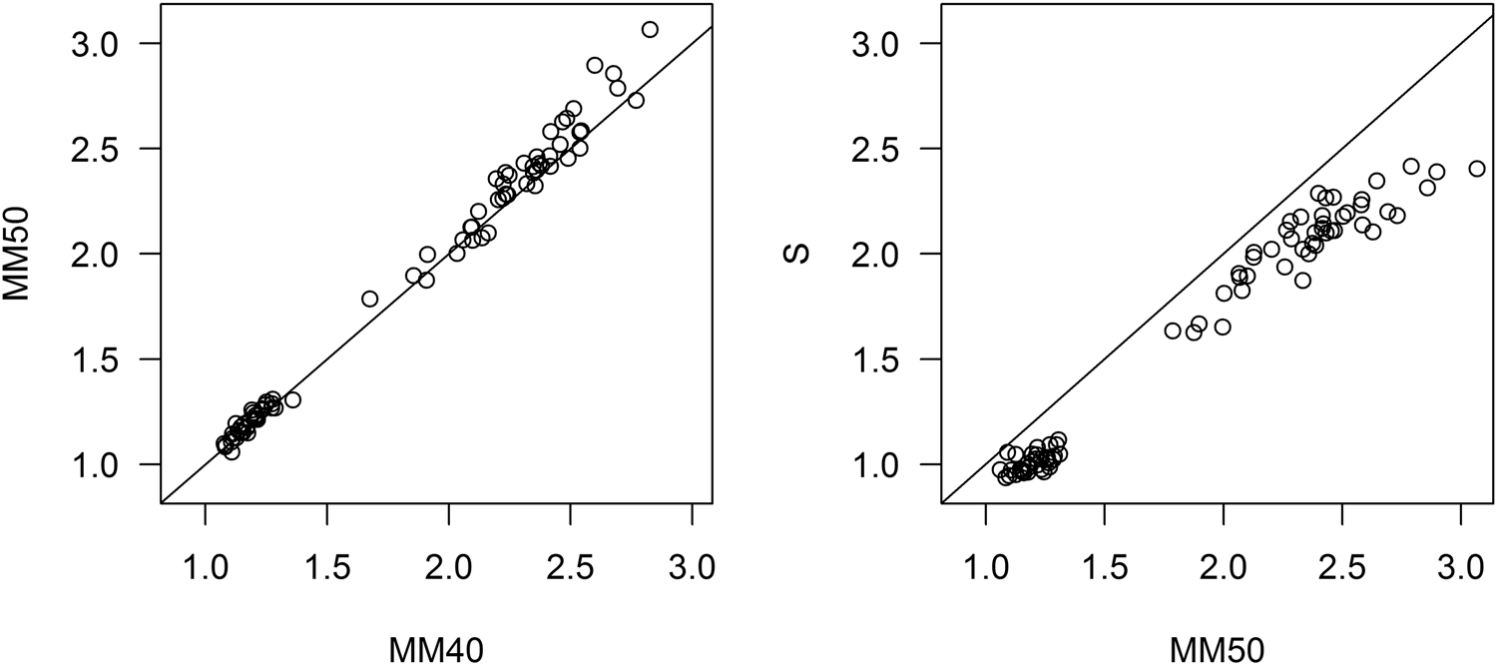
Scatterplots of each of the two steps in the Two-Step pathway: MM40 vs MM50 (change in acquisition), and MM50 vs S (change in method). Each plot includes a 1 = 1 line, to aid comparison.

In Fig. 2 we show scatterplots of each of the two steps in the Two-Step pathway: MM40 vs MM50 (change in acquisition), and MM50 vs S (change in method). In our first step, which corrects differences in acquisition, MM40 (using PiB_40–60_) generally produced comparable or smaller SUVR values than MM50 (PiB_50–70_). In the second step, which corrects differences in processing methods, the Mayo MM50 method generally produced larger values than the standard method S.

**Figure 3:**
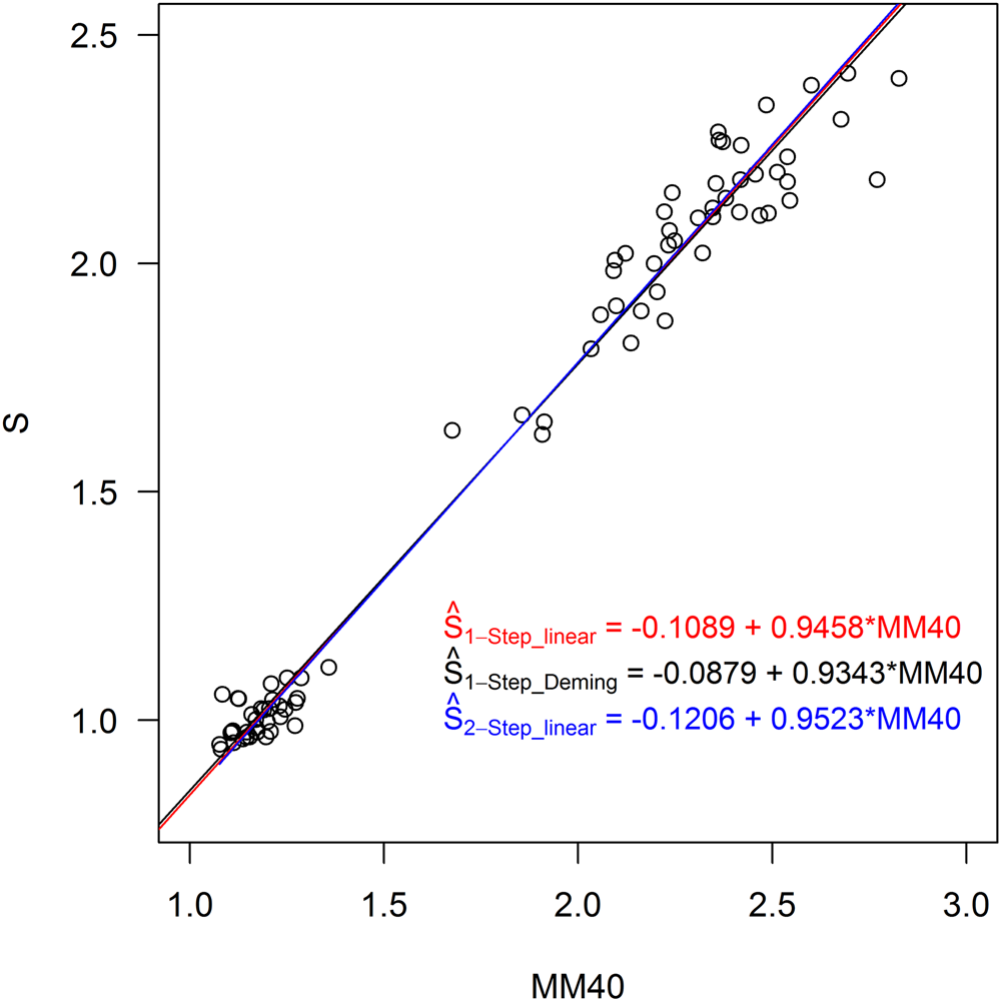
Regression lines computed by using each of the variations in Table 2, shown on the scatterplot between the non-standard method (MM40) and the standard Centiloid PiB method (S). Equations are given in the form in which they would be used to convert MM40 to Ŝ, inverted from their calculated forms that used S as the predictor variable.

In Fig. 3 we show the regression lines created by using each tested regression variant on a scatterplot between the non-standard SUVRs (MM40) and the standard method SUVRs (S). We show the differences between SUVR values using each variant (Ŝ) and the reference values (S) in Table 3. The Ŝ_1-Step_linear_ method’s SUVRs had the smallest mean difference from the reference values, but all methods fell below the 2% threshold^1^ and so are considered valid transformations of the MM40 data to Centiloid units.

**Table 3:** Comparisons between S and calculated S (Ŝ) for each group (YC-0: young controls, the zero-point of the Centiloid scale; and AD-100: subjects with Alzheimer’s disease, the 100-point of the scale), using each of the variations of Ŝ (defined in Table 2).

	YC-0					AD-100				
	Mean (SUVR)	Mean Diff from S (SUVR)	Mean % Diff from S (SUVR)	Mean (CL)	Mean Diff from S (CL)	Mean (SUVR)	Mean Diff from S (SUVR)	Mean % Diff from S (SUVR)	Mean (CL)	Mean Diff from S (CL)
S	1.0095	0.00000	0.00%	0.000	0.000	2.0761	0.00000	0.00%	100.00	0
Ŝ_1-Step_linear_	1.0098	0.00029	**0.03%**	0.027	0.027	2.0759	−0.00022	**−0.01%**	099.98	−0.02042
Ŝ_1-Step_Deming_	1.0172	0.00773	0.76%	0.718	0.718	2.0703	−0.00579	−0.28%	099.46	−0.54286
Ŝ_2-Step_linear_	1.0057	−0.00382	−0.38%	−0.361	−0.361	2.0790	0.00291	0.14%	100.27	0.27258

The variation with the smallest deviation from S is marked in bold.

## Discussion

Overall, there were no qualitative differences in the regression equations produced by variation in how the regression step(s) were performed: regression lines presented the scatterplot in Fig. 3 visually overlap almost completely. The differences between S and Ŝ (Table 3) were all within <2%, which is considered the threshold for transformation acceptability^1^.

From comparing the differences in Table 3, we found that the Ŝ_1-Step_linear_ (one-step, standard linear regression) variant of transforming our non-standard Mayo PiB SUVR method to the Centiloid Standard PiB SUVR scale had the smallest mean differences. However, the differences between transformation methods were negligible and therefore all of these techniques can be considered acceptable. We hypothesize that this is mainly because the dataset contains a large dynamic range and wide separation between the YC and AD groups, providing robustness to differences in regression techniques. Because it is necessary to choose one method, our research group will use the Ŝ_1-Step_linear_ method for our transformations to the Centiloid scale because it had (insignificantly) the smallest mean error.

It is unknown whether our conclusions are specific to our two necessary transformations (PiB_40–60_ to PiB_50–70_, and the Mayo processing method to the Standard processing method). For example, the PiB_40–60_ to PiB_50–70_ conversion uses data that overlaps in the 50–60 minute range, making the samples in this regression less independent than in a transformation across tracers, which would use independent image acquisitions. Therefore, generalizability and confirmation of our findings will rely on future testing by other groups performing different combinations of level-2 transformations. We also did not examine other combinations, such as two-step approaches with Deming regression, nor linear regression approaches using different, non-standard choices for dependent vs. independent variable. Based on our current findings we hypothesize that the differences between these approaches would also be negligible.

## Conclusions

Our data suggests that the numerical differences between Centiloid transformations using one-step and two-step regression approaches, and between standard Linear and Deming regressions, are negligible. All tested variants produced values that were on-average within 1CL of the reference values, and the differences between them also differed by less than 1CL, suggesting that all of these variants produced reasonable transformations to the Centiloid scale.

## Electronic supplementary material

Supplementary Material

## Footnotes

1 ^†^For brevity and for consistency with the Centiloid manuscript, we use the word “standard” to denote the method the scale is based on. We do *not* intend to imply that this specific SPM8-based method is any more common or accepted in the field than others described as “non-standard”.

2 ^‡^Although the Centiloid manuscript is ambiguous about whether mean or median is used in the Standard method, its authors confirmed via e-mail correspondence that it uses the mean value of voxels within each VOI.
