# Supplementary material for: Considerations for Performing Level-2 Centiloid Transformations for Amyloid PET SUVR values
Source: Sci Rep. 2018 May 9;8:7421. doi: 10.1038/s41598-018-25459-9 (PMC5943521; doi:10.1038/s41598-018-25459-9)
Supplement: Supplementary file 1 — Supplementary Material [file 41598_2018_25459_MOESM1_ESM.docx]

Supplementary Material For:
Considerations for Performing Level-2 Centiloid Transformations for Amyloid PET SUVR values

Christopher G. Schwarz^a*^, Nirubol Tosakulwong^b^, Matthew L. Senjem^ac^ , Jeffrey L. Gunter^ac^, Terry M. Therneau^b^, Prashanthi Vemuri^a^, Val J. Lowe^a^, Clifford R. Jack Jr.^a^

^*^Corresponding Author ^a^Department of Radiology, Mayo Clinic and Foundation, Rochester, MN, USA
^b^Department of Health Sciences Research, Division of Biostatistics, Mayo Clinic and Foundation, Rochester, MN, USA
^c^Department of Information Technology, Mayo Clinic and Foundation, Rochester, MN, USA

Correspondence to: Christopher G. Schwarz, Ph.D., Mayo Clinic, Diagnostic Radiology, 200 First Street SW, Rochester, Minnesota, 55905, USA.
E-mail: schwarz.christopher@mayo.edu
Phone: 1 (507) 538-4967
Fax: 1 (507) 284-9778

# Replication of Level-1 Analysis

According to the Centiloid manuscript ^1^, the first step in a level-2 analysis is replication of the level-1 analysis. Specifically, this step tests agreement between our replication of the Centiloid Standard method (MS50) and the published reference values from the Centiloid Standard method (S). We perform this replication step in this section.

For each of the four standard reference VOIs, we performed the standard linear regression (with S as the predictor), and we present the slopes, intercepts, and R^2^ values for each in Table 1.We also provide the scatterplots, with regression lines, for each reference VOI in Figure 1. For each variant, the intercept, slope, and R^2^ values were within the prescribed tolerances for a valid replication (intercept -2 to 2 CL, slope 0.98 to 1.02 CL, R^2^>0.98) ^1^. In Table 2 we present the mean PiB SUVRs for the each group, for S and MS50. All values for the replication (MS50) fit fall within the prescribed tolerances for a valid replication (2% of the values for S). Because all values in Table 1 and Table 2 fall within the prescribed tolerances, we consider our replication valid, thus fulfilling this requirement for a level-2 Centiloid analysis.

Table 1: Agreement of S and MS50 Centiloid values.

| Dataset Stat (Tolerance)  Reference VOI | Slope (0.98 to 1.02) | Intercept (-2 to 2 CL) | R^2^ (> 0.98) |
| --- | --- | --- | --- |
| Whole cerebellum (WC) | 0.9976 | 0.143 | 0.9994 |
| Cerebellar gray (CG) | 0.9978 | 0.125 | 0.9993 |
| Whole cerebellum + brainstem (WCB) | 0.9979 | 0.117 | 0.9995 |
| Pons | 0.9982 | 0.103 | 0.9996 |


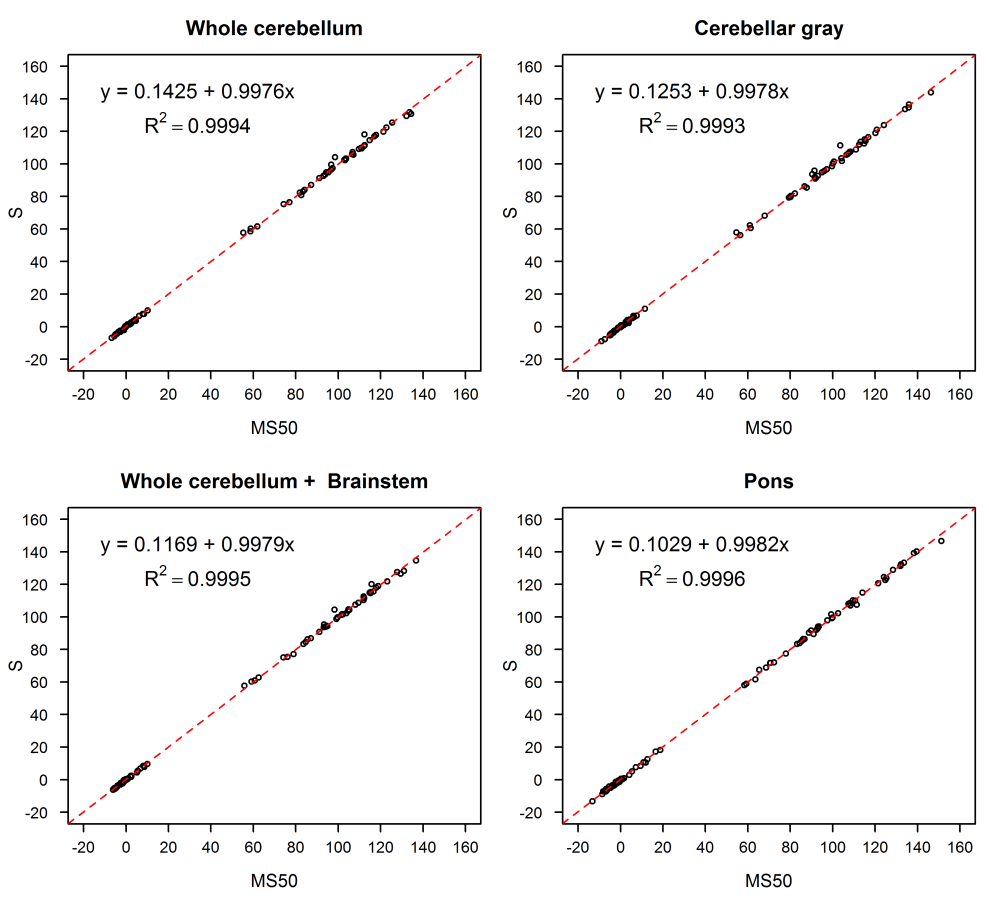


Figure 1: Regression scatterplots for replication of level-1 analysis

Table 2: Mean PiB SUVRs between S and MS50 data.

| Subgroup | YC-0 | | AD-100 | |
| --- | --- | --- | --- | --- |
| Dataset Stat (Tolerance)  Reference VOI | S Mean (±2%) | MS50 Mean (% diff) | S Mean(±%2) | MS50 Mean (% diff) |
| Whole cerebellum (WC) | 1.0095 (0.98931, 1.0297) | 1.0116 (0.21%) | 2.076 (2.0346, 2.1176) | 2.0836 (0.36%) |
| Cerebellar gray (CG) | 1.1702 (1.1468, 1.1936) | 1.1718 (0.14%) | 2.4276 ( 2.379, 2.4762) | 2.4351 (0.31%) |
| Whole cerebellum + brainstem (WCB) | 0.95879 (0.93962, 0.97797) | 0.9613 (0.26%) | 1.962 (1.9228, 2.0013) | 1.9708 (0.45%) |
| Pons | 0.76129 (0.74607, 0.77652) | 0.7598 (-0.19%) | 1.5348 (1.5041, 1.5655) | 1.539 (0.27%) |

# References

1. Klunk, W. E. *et al.* The Centiloid Project: standardizing quantitative amyloid plaque estimation by PET. *Alzheimer’s Dement.* **11,** 1–15 (2015).
